# A Water-Soluble Pro-Drug of Acetaminophen Has Increased Intracellular Delivery of Active Drug and Anti-Cancer Activity in Triple-Negative Breast Cancer and Pancreatic Ductal Adenocarcinoma

**DOI:** 10.3390/cancers18203390

**Published:** 2026-10-20

**Authors:** Allyn Bryan, Rudra Pangeni, Jephte Y. Akakpo, Alex Wendling, Madison Isbell, Rebecca K. Martin, George Weistroffer, Hartmut Jaeschke, Diane Ingabire, Won S. Lee, Anthony C. Faber, Stacey M. Bagby, Todd M. Pitts, Joseph Landry, Bhaumik Patel, Qingguo Xu, Alexander Neuwelt

**Affiliations:** 1 Department of Veterans Affairs, Richmond, VA 23249, USA; allyn.bryan@va.gov (A.B.);; 2 Department of Pharmaceutics, Virginia Commonwealth University, Richmond, VA 23298, USA; rudrapa@pcom.edu (R.P.);; 3 Department of Pharmaceutical Sciences, School of Pharmacy, Philadelphia College of Osteopathic Medicine, Suwanee, GA 30024, USA; 4 Department of Pharmacology, Toxicology and Therapeutics, University of Kansas, Kansas, KS 66160, USA; 5 Department of Microbiology and Immunology, VCU School of Medicine, Richmond, VA 23298, USA; 6 VCU Philips Institute, Virginia Commonwealth University School of Dentistry and Massey Comprehensive Cancer Center, Richmond, VA 23298, USA; 7 Department of Pediatrics, VCU School of Medicine, Richmond, VA 23298, USA; 8 Department of Medical Oncology, University of Colorado, Anschutz Medical Campus, Aurora, CO 80045, USAtodd.pitts@cuanschutz.edu (T.M.P.); 9 Department of Cellular, Molecular and Genetic Medicine, Virginia Commonwealth University, Richmond, VA 23298, USA; 10 Department of Ophthalmology, Department of Biomedical Engineering, Department of Pediatrics, Center for Pharmaceutical Engineering, Center for Drug Discovery, Virginia Commonwealth University, Richmond, VA 23298, USA; 11 Department of Hematology, Oncology and Palliative Care, Virginia Commonwealth University, Richmond, VA 23298, USA

**Keywords:** acetaminophen, propacetamol, pro-drug

## Abstract

**Background/Objectives**. Triple-negative breast cancer (TNBC) and pancreatic ductal adenocarcinoma (PDAC) are two of the deadliest adult cancers. High-dose acetaminophen (AAP) has anti-cancer activity when given in conjunction with CYP2E1 inhibitors to block liver toxicity. However, the administration of high doses of AAP presents practical challenges stemming from limited water solubility, making it difficult to create a concentrated treatment solution in limited volume. Propacetamol (PROP) is a water-soluble AAP pro-drug that has been used clinically as an IV AAP formulation for analgesia. **Methods**. We sought to investigate the pharmacokinetics, safety and anti-cancer activity of PROP in comparison to AAP using transplantable syngeneic models of TNBC and PDAC. **Results**. In vivo, PROP was rapidly converted to AAP within minutes of administration. PROP demonstrated enhanced effectiveness in suppressing tumor growth in the TNBC and PDAC syngeneic murine tumor models compared to AAP. The enhanced efficacy of PROP appears to stem from increased cellular uptake, leading to intracellular AAP concentrations that are 2.7-fold higher within 5 min compared to cells treated with unmodified AAP. PROP demonstrated enhanced modulation of intracellular JAK/STAT signaling, a key mediator of AAP anti-cancer activity, using both murine and humanized models. STAT3 and STAT6 inhibition by PROP led to modulation of macrophage polarization, likely contributing to its anti-TNBC and anti-PDAC activity. While parenteral administration is the preferred route of administration for PROP in anti-cancer indications, we also evaluated the safety and efficacy of oral PROP in comparison to AAP. High-dose PROP demonstrated improved safety following oral administration compared to AAP in the BALB/c murine model while maintaining anti-cancer activity in vivo. **Conclusions**. PROP, an AAP pro-drug, demonstrates enhanced intracellular delivery of AAP and in vivo anti-cancer activity compared to unmodified AAP, setting the stage for investigation as an anti-cancer drug across different indications.

## Simple Summary

We demonstrate that the water-soluble pro-drug propacetamol (PROP) overcomes logistical challenges for the administration of high doses of acetaminophen (AAP) that are needed for anti-cancer activity. Furthermore, we demonstrate that PROP has enhanced anti-cancer activity compared to AAP across multiple syngeneic murine tumor models of triple-negative breast cancer (TNBC) and pancreatic cancer, two difficult-to-treat and common cancers.

## 1. Background

TNBC is the most aggressive and deadly subtype of breast cancer. While hormone-positive breast cancer has benefited from the advent of targeted therapies, TNBC has had minimal benefit to date [1]. Similarly, beyond the recent FDA approval of daraxonrasib for pancreatic cancer [2], there remains a large need to improve pancreatic cancer therapies, with five-year survival of metastatic pancreatic ductal adenocarcinoma (PDAC) still below 15% [3].

Acetaminophen (AAP, Tylenol©) can be administered at high doses (up to 650 mg/kg) safely in mice when combined with CYP2E1 inhibitor drugs—such as fomepizole—to prevent liver toxicity [4]. At these high doses, AAP has substantial anti-cancer activity in a broad spectrum of malignancy models, including melanoma, breast cancer, and lung cancer [4,5,6].

However, the administration of high-dose AAP presents challenges in drug delivery. The limited water solubility of AAP makes it difficult to administer as a concentrated solution in limited volume via IV infusion, and oral administration is impractical. Existing formulations of commercially available liquid AAP for IV infusion (such as Ofirmev ©) are 1 g/100 mL of solution. Prior clinical trials investigating high-dose AAP for the treatment of cancer achieved doses as high as 20 g/m^2^ AAP (about 40 g in an average-sized adult) without liver toxicity [7]. However, 40 g of AAP in liquid form would require about 4 L of IV infusion solution, an unsafe volume to be administered to a human.

AAP is among the most commonly administered drugs in the world, and there have been innumerable attempts to chemically modify AAP’s basic structure to enhance its physicochemical properties [8]. To improve the water solubility of AAP, modifications have included ester- or phosphonate-linked pro-drugs that are readily cleaved in vivo by esterases or phosphatases, respectively, to yield parental drug. These approaches have had some success. For instance, phosphonate-linked pro-drugs of AAP have been reported to have solubilities up to 160 mg/mL, 16-fold higher than unmodified AAP [8]. Therefore, water-soluble pro-drugs could conceivably be used to administer high doses of AAP in volumes that are lower and more feasible, rapidly being converted to unmodified AAP in vivo by circulating phosphatases.

The most commercially successful AAP pro-drug to date is propacetamol (PROP). PROP is a highly water-soluble, ester-based pro-drug that has been marketed for many years in Europe and Asia as a parenteral (IV) formulation of AAP. Importantly, a large Cochrane Review of 7200 patients documented the safety of PROP in the clinical setting [9]. Indeed, PROP exhibits a similar safety and analgesic efficacy profile as AAP. However, PROP is associated with a higher incidence of infusion-related pain, which is likely due to the acidic pH (approximately 3.5) of its formulated preparations [10]. To overcome logistical limitations for the administration of high-dose AAP, in the present manuscript, we investigated the safety and PK properties of PROP and evaluated the feasibility and efficacy of water-soluble, highly concentrated PROP for the treatment of murine syngeneic models of TNBC (which has already been shown to be highly sensitive to AAP in murine syngeneic models [5]) and PDAC (a relatively common malignancy with few effective therapies).

## 2. Methods

### 2.1. Drugs and Chemicals

Two pro-drugs of AAP (AAP phosphonate pro-drug (Supplemental Figure S1) and PROP (Supplemental Figure S1) (purity 99% for both drugs) were custom synthesized by WuXi Biologics (San Diego, CA, USA) and confirmed by NMR (Supplemental Figure S1). Acetaminophen (purity 99%), N-acetylcysteine (NAC, purity > 98%), and 2-hydroxypropyl-β-cyclodextrin (HBC, purity > 98%) were purchased from Sigma (St. Louis, MO, USA). HBC was used to solubilize AAP. Fomepizole (4-methylpyrazole, purity 99%) was purchased from SelleckChem (Houston, TX, USA) and diluted to 20 mg/mL in 5% glucose water. AAP was diluted in 5% glucose and 10% HBC solution to a concentration of 33 mg/mL for administration. NAC was diluted to a concentration of 20 mg/mL and administered in 5% glucose solution. When combined with NAC and fomepizole, the final pH of the AAP solution was 7.5. The typical volume of the AAP/NAC/fomepizole solution is 790 µL for a 650 mg/kg treatment in a 30 g mouse. Gemcitabine (Infugem 10 mg/mL) was obtained from the Richmond VAMC clinical pharmacy (Richmond, VA, USA). PROP was formulated at 80 mg/mL in 5% glucose in water, and the pH after combination with NAC/fomepizole was 4.5. The typical volume is 570 µL in a 30 g mouse for 1000 mg/kg PROP treatment. PROP is water soluble and can be prepared in physiologic saline for short-term administration; however, because it undergoes hydrolysis in aqueous solution, particularly at higher temperatures and near-neutral pH [11], the formulation was prepared immediately before use.

### 2.2. Cell Lines

4T1 and AT-3 cells were a generous contribution from collaborator Joseph Landry (Department of Genetics, VCU, Richmond, VA, USA). PAN02 cells were acquired from the DCTD tumor repository. CPK tumor cells were obtained from Anthony Faber. KPC lines are from the laboratory of Todd Pitts. Cell lines were routinely validated using morphology and were checked for mycoplasma using a commercial kit (American Type Culture Collection, Manassas, VA, USA). Authenticity of lines was confirmed using short-tandem repeat analysis.

### 2.3. Mouse Studies

BALB/c mice and C57bl/6 mice aged 8–12 weeks old were obtained from Jackson Labs (Bar Harbor, ME, USA) and bred in-house. Humanized mice were created by transplanting purified CD34^+^ hematopoietic stem cells derived from human cord blood into immunodeficient FLT3 NSG mice (NOD.Cg-Flt3em2Mvw Prkdcscid Il2rgtm1Wjl Tg(FLT3LG)7Sz/SzJ), as we have previously reported [12]. Mouse experiments were conducted at the Richmond VA Medical Center, with approval of the IACUC committee (protocol #1789596). KPC experiments were performed at the University of Colorado Anschutz Medical Campus with IACUC approval (protocol #00021). All mice were analyzed, and the results are shown; no exclusion criteria were used once the study was initiated. Humane endpoints for the mice included tumor size > 1500 mm^3^ and weight loss of 20% or more from baseline, which resulted in the mice being euthanized. To minimize potential bias, including treatment order effects, mice were randomized to ensure comparable average tumor volumes across all groups at treatment initiation. Mice with poor tumor formation, indicated, for instance, by linear tumors or tumors that are too superficial to the skin, were excluded. Blinding was not used because the technicians had to be aware of the treatment groups to treat the mice. Anesthesia with isoflurane was used to reduce animal pain during tumor implantation.

### 2.4. In Vivo Tumor Models

Orthotopic 4T1 and AT-3 tumors were created by injecting tumor cells (10^6^ cells in 50 µL solution of 50% media and 50% Matrigel cat # 356234 from Corning, Corning, NY, USA) directly into the breast tissue of female BALB/c mice and female C57bl/6 mice, respectively. PAN02, KPC and CPK tumor models were created by subcutaneously injecting tumor cells (10^6^ cells in 50 µL solution of 50% media and 50% Matrigel from Corning) into the flanks of C57bl/6 mice (both males and females, 8–12 weeks old were used). Treatment was initiated once tumors were palpable and approximately 50 mm^3^; more exact tumor sizes at treatment initiation are provided in the Figure legends. Treatment for all tumor models was IP (intraperitoneal) 2×/week, unless otherwise indicated. Tumor length and width were measured using a digital caliper every three days, and the tumor volume was calculated as: ½ (Length × Width^2^), where the length was the largest tumor diameter. Blood chemistry analyses, including ALT and BUN, were performed by the Richmond VAMC clinical laboratory.

### 2.5. Bone Marrow Macrophage Differentiation

The procedure for generating bone marrow-derived macrophages (BMDMs) has been previously published [5,6]. M1 macrophages are generated using overnight incubation with LPS (100 ng/mL, cat # 00-4976-93, Invitrogen, Carlsbad, CA, USA) and interferon gamma (20 ng/mL, cat 315-05-500UG, Invitrogen). M2 macrophages are generated using overnight incubation with IL-4 (20 ng/mL, cat 214-14-20UG, Invitrogen).

### 2.6. In Vivo Pharmacokinetics (PK)

To evaluate the PK parameters, C57bl/6 mice (male, age 8–12 weeks) were administered PROP (1000 mg/kg IP) or AAP (500 mg/kg IP) along with N-acetylcysteine (NAC, 100 mg/kg) and/or fomepizole (30 mg/kg) as indicated in the respective figure legends. Subsequently, mice were euthanized, and blood samples were collected by cardiac puncture at predetermined intervals. The blood was immediately centrifuged (2500 *g*, 15 min, 4 °C), and the serum was separated and stored at −80 °C until analysis.

Serum concentrations of AAP and PROP were quantified using a protein precipitation-based extraction method followed by LC–MS analysis. In brief, 50 µL of defrosted serum sample was spiked with 20 µL of paracetamol d4 internal standard (10 µg/mL, cat #LGCAMP0042.80-01, LG Chemicals, Atlanta, GA, USA). Proteins were precipitated by adding 600 µL of ice-cold acetonitrile, followed by vortex mixing and centrifugation at 10,000 *g* for 5 min at 4 °C. The resulting supernatant was transferred to a glass vial and evaporated to dryness using a sample concentrator dry-block (Techne, Cambridge, UK) at 50 °C. The residue was reconstituted with 150 μL of acetonitrile/water (90:10, *v*/*v*). Although PROP was assayed, it was below the limit of detection (10 ng/mL) at all time points, presumably because PROP was rapidly converted to AAP. The serum concentration of AAP was determined using an LC–MS system (LC-MS-2020) (Shimadzu Corporation, Kyoto, Japan) equipped with a Phenomenex Kinetex C18 column (100 × 2.1 mm, 1.7 μm, PFP 100 Å). Chromatographic separation was achieved under isocratic conditions with a mobile phase consisting of water with 0.1% formic acid and acetonitrile with 0.1% formic acid (40:60, *v*/*v*) at a flow rate of 0.3 mL/min. A 10 μL sample was injected, and AAP was measured in positive ionization mode using an electrospray ionization interface. The following parameters were optimized for AAP analysis: interface temperature, 350 °C; DL temperature, 300 °C; nebulizing gas flow, 1.2 L/min; drying gas flow, 12 L/min. For cellular permeability assays, tumor cells were treated with 1 mM AAP or PROP. The cells were collected at the indicated time point, lysed, and intracellular AAP levels measured using LC–MS. AAP metabolites (AAP-glucuronide, etc.) were measured by co-author JA using his published methodologies at the University of Kansas [13].

### 2.7. In Vitro Assays

Immune-blotting and qPCR were performed using established protocols. Western blotting was conducted via lysing cells in RIPA buffer with protease and phosphatase inhibitors. Samples were then heated to 70 °C for 10 min. Fifty micrograms of sample were added per well. Transfer to a PVDF membrane was performed after activation with methanol. The membrane was then washed with TBST and blocked with 5% BSA in TBST for 1 h at RT. The membrane was then washed with TBST. The membrane was then incubated with the primary antibody overnight. The membrane was washed 3× with TBST. Incubation with HRP secondary antibody was 1 h at RT. The membrane was washed 6× with TBST. Signal was detected using Super Signal West PICO Plus (Thermo Scientific, Walham, MA, USA). The membrane was imaged using the iBright Imaging System (Invitrogen, Carlsbad, CA, USA). Densitometric analysis of Western blots was performed using UN-SCAN-IT software (Silk Scientific, Provo, UT, USA, Version 7.0). The primer sequences used for the RNA studies were described previously [6,14] and are listed in Supplemental Table S1. Cells were lysed using Trizol (Invitrogen) according to the manufacturer’s protocol. RNA was quantified on a NanoDrop (Thermo Scientific). The BioRad iScript cDNA (Hercules, CA, USA) synthesis kit was used to make cDNA with 1 µg of RNA per sample. Forty nanograms of cDNA per reaction were used for qPCR using the QuantaBio Perfecta SYBR Green Fastmix. Samples were analyzed by Quant Studio 3 (Applied Biosystems, Foster City, CA, USA) and normalized using actin. The original blot can be found in the Supplementary Materials.

### 2.8. ImageStream and Flow Cytometry Analysis

Humanized mice were treated as indicated. The mice were euthanized 30 min after treatment. Peripheral blood white blood cells were isolated and fixed using the manufacturer’s standard protocols (BD Phosflow^TM^). In brief, BD Lyse/Fix buffer (cat# 558409, BD Biosciences, San Jose, CA, USA) was heated to 37 °C, added to the whole blood at a 20:1 ratio, and incubated for 10 min. Cells were then permeabilized with Perm Buffer III (Cat#: 558050, BD) on ice for 30 min, washed and stained with pSTAT3 antibody FITC-conjugated (cat#11-9033-42, Invitrogen) and CD11b antibody PerCP-Cy 5.5-conjugated (Cat# 101228, BioLegend, San Diego, CA, USA). Samples were analyzed using an Image Stream imaging flow cytometer or an LSRX20 Fortessa flow cytometer (BD Biosciences, San Jose, CA, USA) according to standard protocols. Image Stream data were analyzed using IDEAS software (CYTEK, Fremont, CA, USA, Version 6.3).

### 2.9. Statistical Analysis

Statistical analyses were performed using GraphPad Prism (GraphPad Software, San Diego, CA, USA, Version 11). Comparison between two groups was conducted using the unpaired Student’s t-test. Tumor growth data were analyzed using two-way ANOVA followed by Bonferroni’s multiple comparison post hoc test. *p* < 0.05 was considered statistically significant. For tumor efficacy studies, sample size calculations were performed assuming a two-sided alpha = 0.05 and power = 0.9, indicating that 7 mice per group were required to detect a minimum 33% difference in tumor size compared to the control group. Mouse number was reduced based on empirical observations if fewer mice would be needed to achieve significance. Error bars represent standard deviation unless otherwise indicated.

## 3. Results

### 3.1. Propacetamol Has Increased Anti-Cancer Activity In Vivo Compared to Acetaminophen

**Figure 1:**
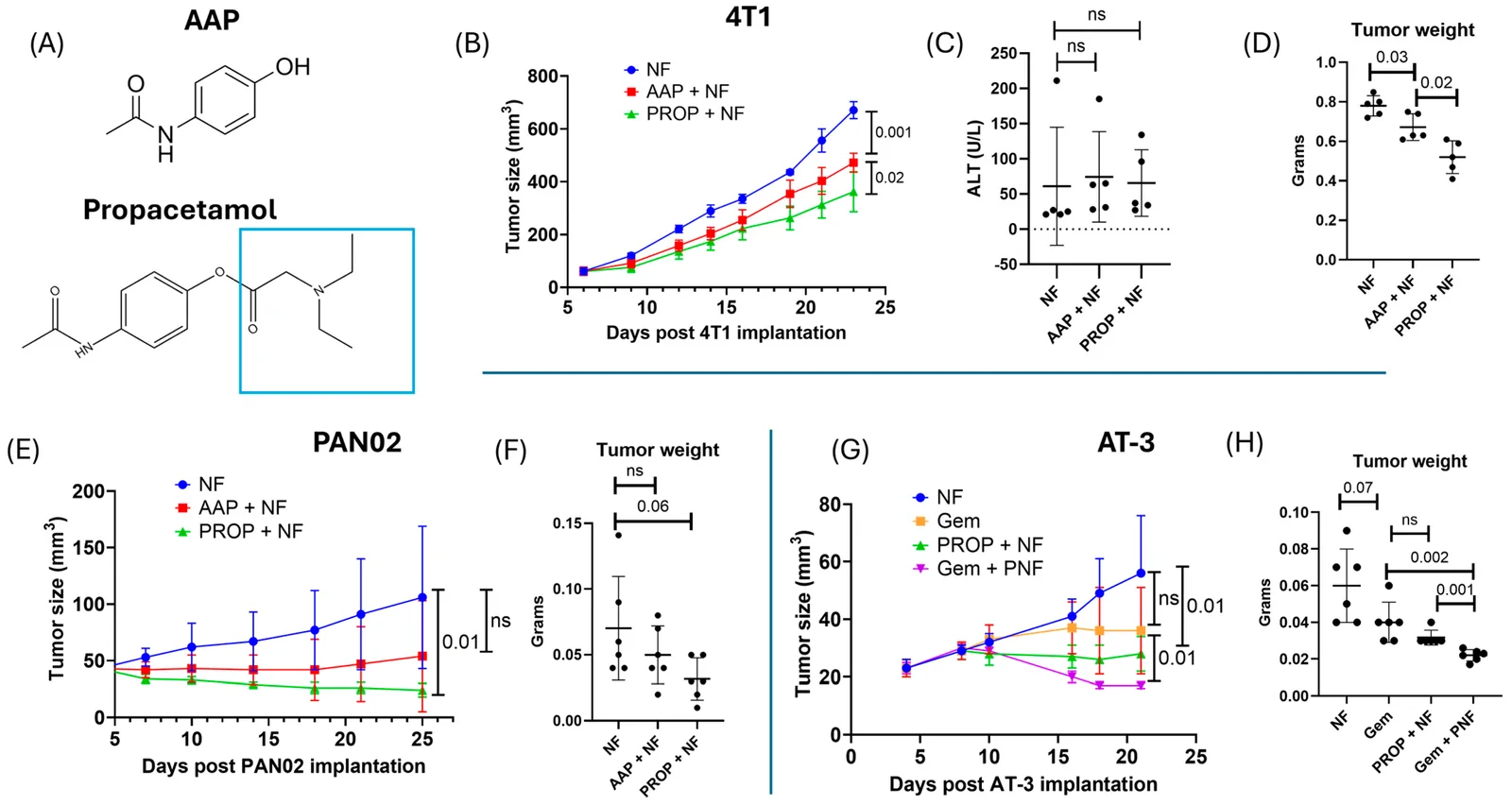
Propacetamol (PROP) exhibits greater antitumor activity than acetaminophen (AAP) in vivo. (**A**) Chemical structures of unmodified AAP and PROP. The pro-drug moiety of PROP is highlighted in the blue box. (**B**) Tumor growth curves of orthotopic 4T1 tumors in female BALB/c mice treated with PROP (1300 mg/kg), AAP (650 mg/kg), fomepizole (F, 30 mg/kg), and N-acetylcysteine (N, 100 mg/kg) by intraperitoneal (IP) injection twice weekly, as indicated. N = 5 mice per group. At the conclusion of the experiment, mice were euthanized, and serum was analyzed for ALT (**C**) and tumor weights were measured (**D**). Average tumor size at treatment initiation was 60 mm^3^. (**E**) Tumor growth curves of subcutaneous PAN02 tumors grown in C57bl/6 mice treated with vehicle (NF), AAP or PROP. Treatment was initiated when tumors were on average 43 mm^3^ and administered as described in (**B**), except that PROP was administered at 1000 mg/kg and AAP at 500 mg/kg. N = 6 mice per group. (**F**) At the end of the experiment, mice were euthanized, and tumor weights were measured. (**G**) Tumor growth curves of mice implanted orthotopically with AT-3 tumors and treated; first treatment when the average tumor was 29 mm^3^, with PROP (P) 1000 mg/kg IP 2×/week along with NF and/or gemcitabine 80 mg/kg IP weekly as indicated. (**H**) Tumor weights at the end of the experiment. N = 6 mice per group. The level of significance is shown, and error bars represent standard deviation. ns = not significant.

PROP is a highly water-soluble (>100 mg/mL), ester-based pro-drug of AAP that is rapidly hydrolyzed to AAP in vivo by plasma and cellular esterases [15]. Because PROP is unstable at neutral pH, it requires preparation immediately before administration, as described in the Methods section. To compare the antitumor efficacy of PROP and AAP, we chose TNBC, as it is a highly aggressive form of breast cancer with a lack of effective therapies. Mice with orthotopically implanted 4T1 TNBC tumors were treated with vehicle, AAP, or PROP. An equimolar dose of drugs was administered to the AAP and PROP groups, though a larger mass of PROP was administered due to its higher molecular weight (accounting for the pro-drug moiety). PROP exhibited greater antitumor activity than unmodified AAP, resulting in a significantly greater reduction in tumor growth. At the end of the study, the mean tumor volumes were 671 ± 32 mm^3^ for the vehicle group, 471 ± 36 mm^3^ for the AAP group, and 362 ± 76 mm^3^ for the PROP group (*p* < 0.05 between AAP and PROP cohorts). Neither AAP nor PROP led to liver toxicity at the doses tested (Figure 1A–D).

In the PAN02 syngeneic PDAC model, IP administration of PROP significantly reduced tumor growth compared to the vehicle control. In contrast, AAP administered at an equimolar dose showed a non-significant trend towards tumor inhibition relative to vehicle (Figure 1E,F).

Because gemcitabine is a foundational chemotherapy backbone for TNBC and PANC [16], we asked whether PROP administered together with gemcitabine would result in enhanced efficacy relative to either single agent. In the AT-3 triple-negative breast cancer model, it was demonstrated that PROP was highly active as monotherapy, leading to approximately 50% tumor size reduction compared to the vehicle control (NAC/fomepizole alone), tumor size 56 ± 20 mm^3^ for vehicle vs. 28 ± 6 mm^3^ for propacetamol. Furthermore, gemcitabine administered in combination with PROP led to increased tumor shrinkage (17 ± 1 mm^3^) compared to gemcitabine alone (36 ± 15 mm^3^, Figure 1G,H, *p* < 0.05), thus demonstrating improvement over an established standard of care treatment for breast cancer patients. Mice tolerated all treatments well, with no significant weight loss observed throughout the study (Supplemental Figure S2).

**Figure 2:**
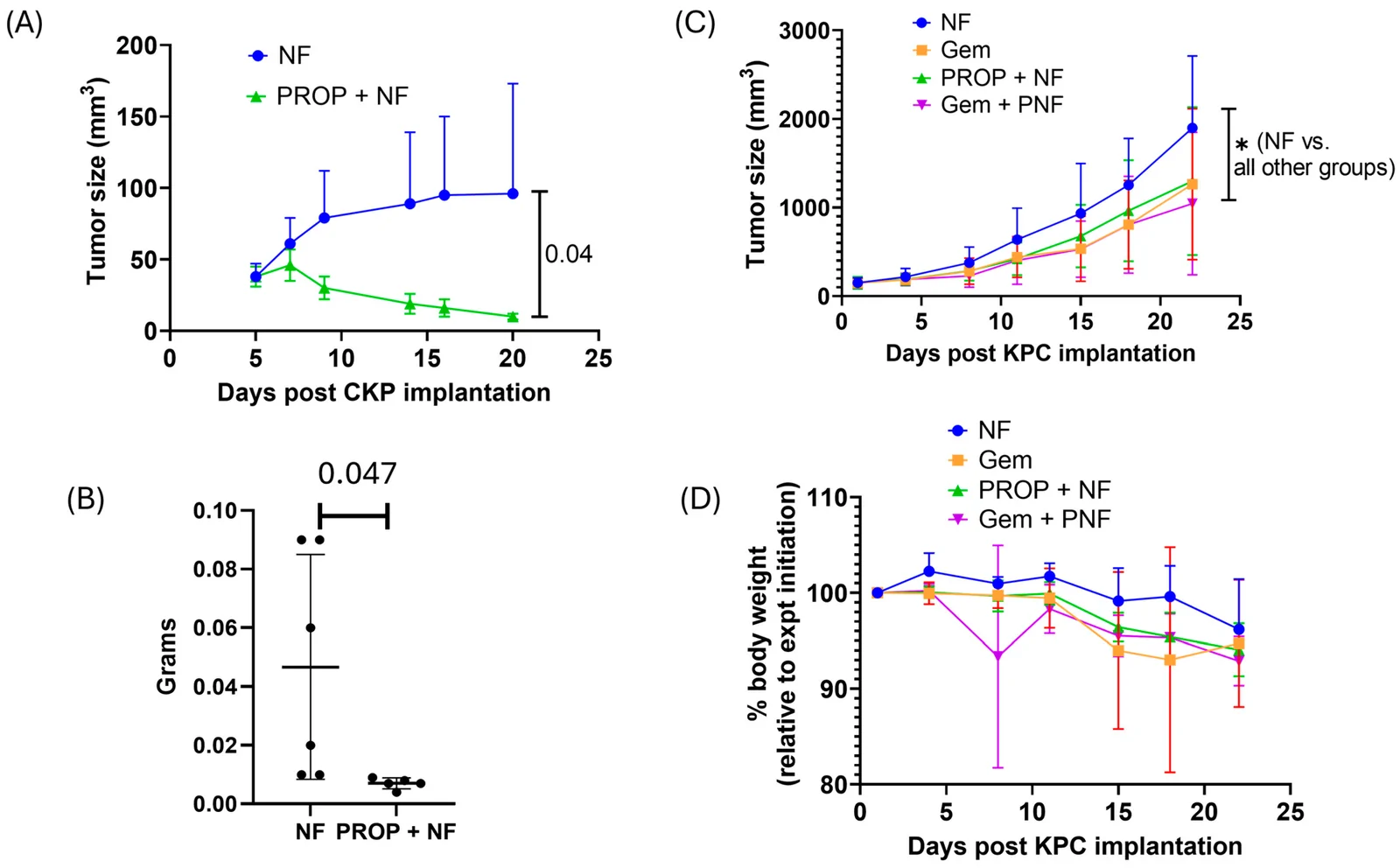
Propacetamol is active in murine syngeneic models of pancreatic cancer. (**A**,**B**) CKP pancreatic cancer cells were implanted in mice, and treatment was initiated once tumors were palpable. Treatments were PROP (1000 mg/kg), fomepizole (F, 30 mg/kg), and N-acetylcysteine (N, 100 mg/kg) twice per week IP. Tumor size was monitored during the experiment (**A**), and tumors were weighed at the end of the experiment. (**B**) N = 6 for NF treatment and 5 for PROP NF condition. (**C**,**D**) KPC tumors were implanted in the bilateral flanks of syngeneic mice and treated with PROP, F, and/or N as indicated. Additionally, mice were treated with gemcitabine (Gem, 80 mg/kg IP weekly) as indicated. Tumor size was monitored with digital calipers (**C**), and mice body weight was recorded (**D**). There was no significant difference in body weights among the various treatment groups. * *p* < 0.05; exact *p* values are also shown. Error bars represent standard deviation.

Due to the substantial activity of PROP in the PAN02 model, we sought to confirm efficacy in additional pancreatic cancer syngeneic models. Here, we employed the CKP PDAC model, a murine syngeneic pancreatic cancer model driven by mutant KRAS [17]. We additionally performed experiments (conducted in an independent laboratory under the direction of co-author TMP) with the widely used KPC model. PROP was highly active in the CKP syngeneic mouse pancreatic cancer model (Figure 2), resulting in a 90% reduction in tumor size compared to vehicle-treated tumors over 15 days of treatment. PROP was also active in the KPC model, demonstrating a similar level of activity as gemcitabine (final tumor sizes 1898 ± 813 mm^3^ for NF; 1263 ± 852 mm^3^ for gemcitabine; and 1297 ± 836 mm^3^ for PROP), a standard first-line therapy in pancreatic cancer (Figure 2C). PROP combined with gemcitabine led to a non-significant trend towards the smallest tumors among the various treatment groups (final tumor size 1045 ± 806 mm^3^) (Figure 2C). On average, all mice in the experiment lost a modest amount of weight (without statistically significant changes between the groups), suggesting the treatments were well tolerated (Figure 2D). Thus, in three representative models of PDAC (PAN02, CKP and KPC) and two representative TNBC models (4T1 and AT3), we demonstrate that PROP has anti-cancer activity.

We also evaluated the antitumor efficacy of a previously described highly water-soluble phosphonate-based AAP pro-drug in the 4T1 model. This pro-drug has a water solubility of approximately 160 mg/mL, compared with 10 mg/mL for the parent compound, AAP [8]. Despite its improved aqueous solubility, the phosphonate-based pro-drug was less effective in reducing tumor growth compared to parental AAP (Supplemental Figure S3). As a result, PROP was selected as the lead water-soluble AAP pro-drug for all subsequent studies.

### 3.2. Propacetamol Has Increased Intracellular Delivery of Active Drug Compared to Acetaminophen

**Figure 3:**
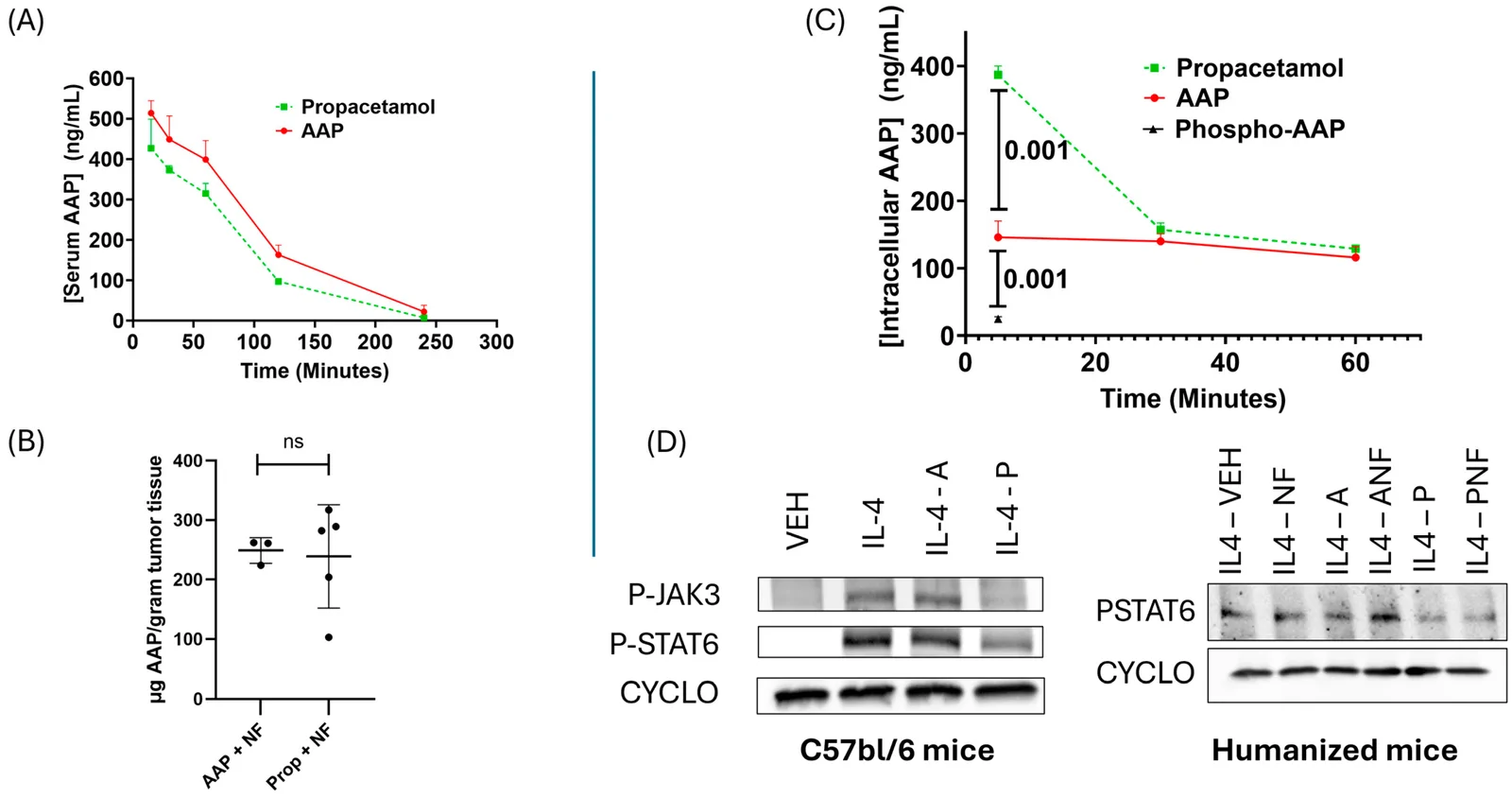
Propacetamol (PROP) pharmacokinetics and conversion to acetaminophen (AAP). (**A**) PROP (P, 1000 mg/kg IP) and AAP (A, 500 mg/kg IP) were administered along with N-acetylcysteine (N, 100 mg/kg) and fomepizole (F, 30 mg/kg). All treatments were administered IP. Serum AAP concentrations were measured at the indicated time points. PROP remained below the limit of detection at all time points, indicating rapid conversion to AAP in vivo. N = 3 mice per time point. (**B**) 4T1 tumor-bearing mice were treated with AAP or PROP IP. Tumors were harvested 30 min after treatment; AAP concentrations were quantified by LC–MS. N = 3 for AAP and n = 5 for PROP biological replicates. (**C**) 4T1 cells were treated with 1 mM AAP, PROP, or phospho-AAP in vitro. At the indicated time points, cells were lysed, and intracellular AAP concentrations were quantified using LC–MS. Only a single time point was obtained for phospho-AAP because this molecule has previously been shown to exhibit inferior anti-cancer activity (as shown in Supplemental Figure S3). N = 3 biological replicates per condition shown. (**D**) BMDM from C57bl/6 mice were pre-treated with vehicle or 3 mM AAP or PROP for 30 min. The macrophages were then stimulated for 30 min with IL-4 (20 ng/mL) as indicated prior to collection for Western blot. The experiment was repeated, but using BMDM derived from mice with a humanized immune system. ns = not significant.

To better understand the underlying mechanisms for the increased antitumor activity of PROP, we analyzed its pharmacokinetic profile. Following IP administration, serum AAP levels were consistently, albeit modestly, lower when PROP was administered relative to AAP. Using LC-MS, we also assayed levels of PROP (in addition to AAP) in the serum. PROP levels remained below the limit of detection (10 ng/mL) at all time points, suggesting rapid esterase-mediated cleavage of the pro-drug moiety and conversion to AAP in vivo (Figure 3A).

We next evaluated the delivery of AAP to the tumor. Tumor-bearing mice were treated with AAP versus PROP, and tumors were harvested 30 min after treatment for quantification of AAP concentration. Tumor AAP levels were comparable between the two treatment groups, with concentrations of 249 ± 22 µg/g tumor following AAP administration and 239 ± 87 µg/g tumor following PROP administration (Figure 3B). These assays measured AAP levels in bulk tumors and did not reveal information on intracellular levels. To assess intracellular levels, in vitro assays were performed due to the complexity of such assays in vivo (intracellular assays of ex vivo tumors require creation of single-cell suspensions utilizing various buffers and collagenases that will alter intracellular concentrations).

4T1 tumor cells were treated in vitro with AAP, PROP, or AAP phosphonate pro-drug. At various time points, cells were lysed, and intracellular AAP concentrations were quantified using LC–MS. At 5 min, it was demonstrated that PROP led to 2.7-fold higher intracellular levels of AAP compared to parental AAP. On the other hand, AAP phosphonate treatment resulted in markedly lower intracellular AAP levels (83% lower with AAP phosphonate compared to unmodified AAP, Figure 3C). These results suggest rapid intracellular uptake of PROP as well as conversion to AAP; intracellular PROP levels were undetectable in cells treated with PROP. The intracellular AAP levels at 30 min and 1 h after treatment were similar whether the cells were treated with PROP or parental AAP (Figure 3C).

To assess the therapeutic relevance of the rapid intracellular uptake of PROP, Western blots were performed assaying STAT6 phosphorylation. We have previously demonstrated that AAP functions as an inhibitor of STAT6 phosphorylation [6], which prevents “M2” polarization of macrophages. In those studies, a 6 h incubation of AAP was used. However, considering that in humans AAP has a half-life of about 4 h [7], shorter incubations may be more physiologically relevant. We thus evaluated whether AAP or PROP could inhibit STAT6 phosphorylation with only a 30 min pre-incubation. It was shown that PROP, but not AAP, led to substantial inhibition of STAT6 phosphorylation with a 30 min pre-incubation, highlighting the significance of PROP’s increased intracellular delivery of AAP (Figure 3D, Supplemental Figure S4). These results were obtained in bone marrow-derived macrophages, both from C57bl/6 mice and mice with a humanized immune system.

### 3.3. Propacetamol Leads to “M1” Activation of Bone Marrow-Derived Macrophages

**Figure 4:**
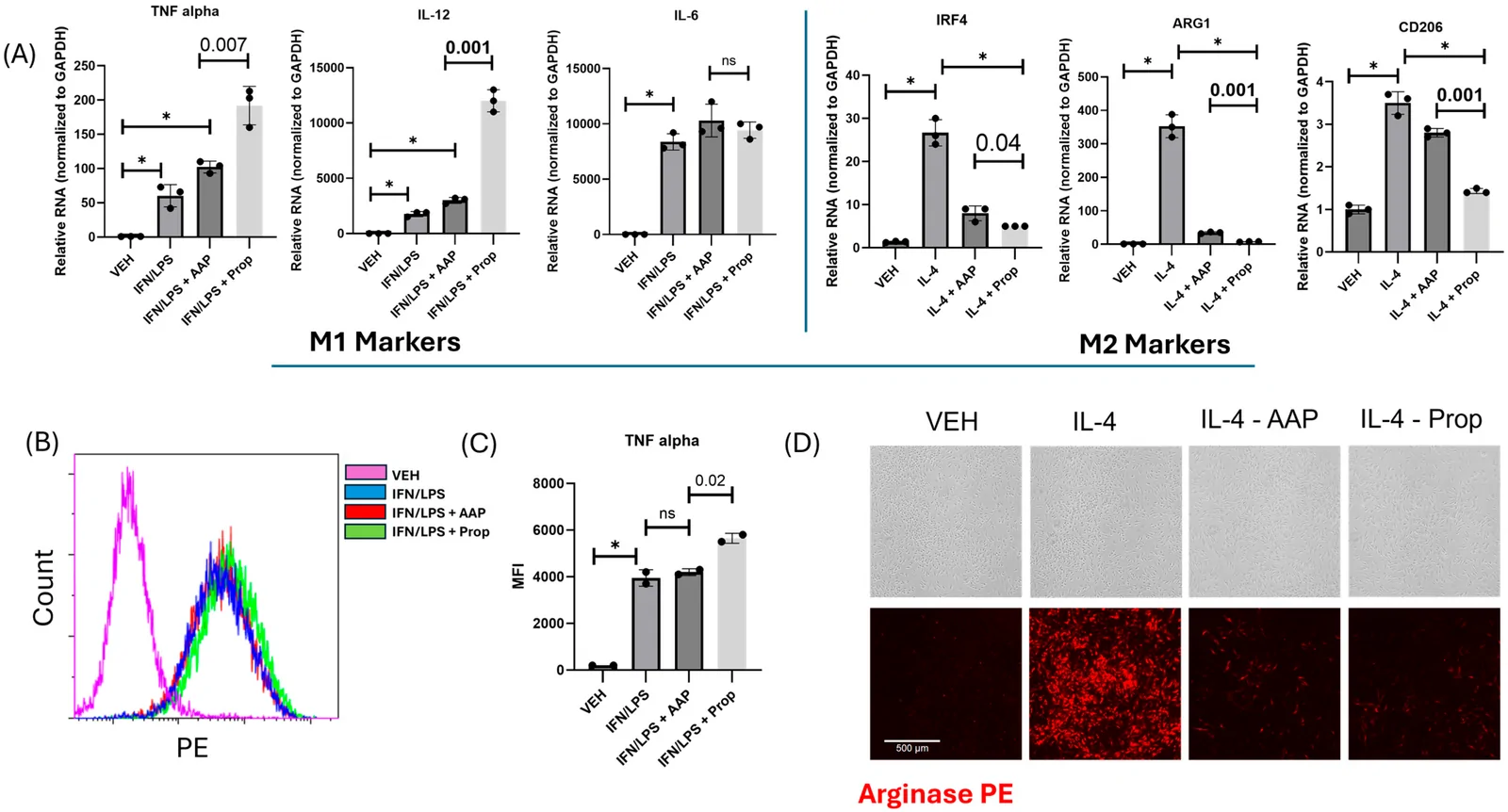
Propacetamol (PROP) leads to “M1” activation of bone marrow-derived macrophages. (**A**) Quantitative PCR of “M1” and “M2” associated genes, evaluated 4 h after treatment of bone marrow-derived macrophages from C57bl/6 mice with vehicle, PROP (1 mM), or acetaminophen (AAP, 1 mM). Macrophages were stimulated with cytokines as indicated. N = 3 technical replicates are shown. (**B**) Same methodology as in (**A**); however, the BMDMs were treated for 4 h as indicated, along with brefeldin A, and intracellular flow cytometry was performed. Representative plots are shown. (**C**) Graph of 2 technical replicates per condition of flow cytometry data shown in (**B**). (**D**) C57bl/6 BMDM were treated overnight with AAP (1 mM), PROP (1 mM) or IL-4 (20 ng/mL). The following day, BMDMs were fixed, permeabilized, and stained with PE-conjugated arginase I antibody. Cells were visualized with a fluorescence microscope (**below**) or light microscope (**above**). * *p* < 0.05, exact *p*-values also shown. ns = not significant.

Having shown that PROP inhibits STAT6 phosphorylation (a regulator of macrophage differentiation [18]), we next evaluated the effects of PROP on “M1” and “M2” markers at the RNA and protein levels. PROP led to a marked increase in the expression of TNF alpha and IL-12, both key cytokines secreted by activated “M1-type” macrophages. On the other hand, PROP led to profound inhibition of “M2-type” genes, including IRF4, ARG1, and CD206. At the protein level, PROP increased production of TNF alpha by BMDMs, as determined by flow cytometry, whereas AAP did not. Both PROP and AAP blocked expression of arginase, a key immunosuppressive marker of “M2” macrophages, as demonstrated using fluorescent microscopy (Figure 4).

### 3.4. Propacetamol Is a STAT3 Inhibitor

**Figure 5:**
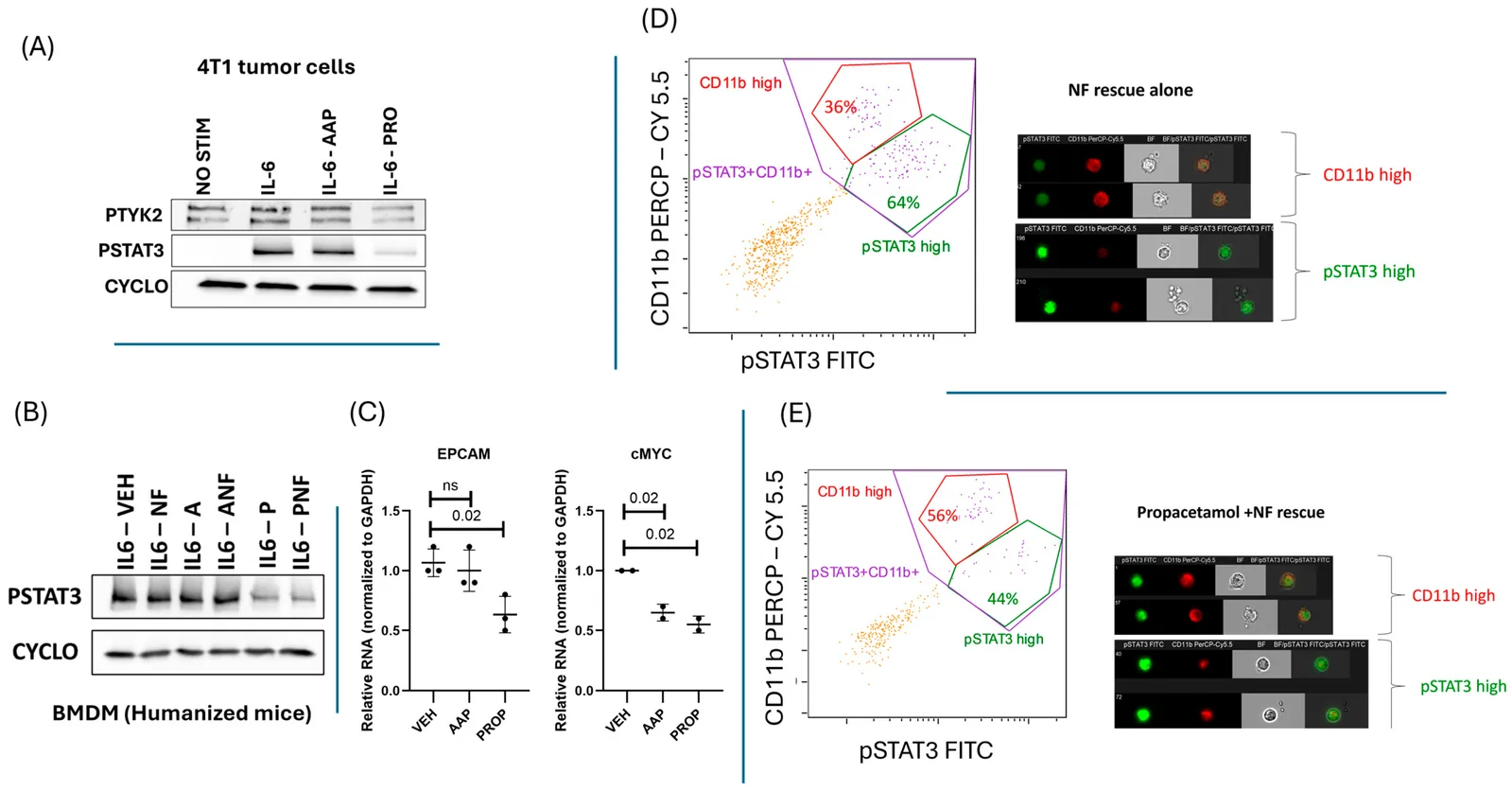
Propacetamol (PROP) functions as a STAT3 inhibitor. (**A**) 4T1 tumor cells were pre-treated with PROP (PRO, 3 mM) or AAP (3 mM) for 30 min prior to stimulation with IL-6 for 15 min. Cell lysate was analyzed with immunoblotting. (**B**) Bone marrow-derived macrophages (BMDMs) were obtained from humanized mice. The BMDMs were treated with AAP or PROP (P, 3 mM) for 30 min with or without human IL-6 (20 ng/mL) prior to collection and analysis by Western blot. (**C**) Quantitative PCR of STAT3-responsive genes, evaluated 4 h after treatment of MDA-MB-436 human breast cancer cells with vehicle, PROP (1 mM) or AAP (1 mM). Data points shown are technical replicates (N = 2–3 per condition). (**D**,**E**) Non-tumor-bearing humanized mice were treated with N-acetylcysteine (N, 100 mg/kg) and fomepizole (F, 30 mg/kg) without (**D**) or with (**E**) PROP (1000 mg/kg). All treatments were administered IP. Thirty minutes later, the mice were sacrificed. The peripheral blood was stimulated for 15 min with IL-6. The red blood cells were lysed, and the remaining cells were analyzed with phospho-flow using standardized protocols prior to analysis using Image Stream flow cytometry. Representative images are also shown. *p* values are shown. ns = not significant.

In addition to blocking STAT6 phosphorylation, we have also shown that AAP functions as a STAT3 inhibitor, which leads to anti-cancer stem cell activity [14]. We thus evaluated the effect of PROP on STAT3 phosphorylation in comparison to AAP with a 30 min pre-incubation time in 4T1 tumor cells. We demonstrated that PROP, but not AAP, inhibited STAT3 phosphorylation under these conditions, possibly a result of decreased intracellular delivery of AAP (Figure 5A, Supplemental Figure S4). PROP similarly inhibited STAT3 phosphorylation in bone marrow-derived macrophages from a humanized mouse (Figure 5B), a relevant finding because STAT3 can also mediate “M2” polarization of macrophages [19]. The pSTAT3 inhibition of PROP was not reversed in vitro when combined with NAC and fomepizole, the rescue agents used to prevent toxicity (Figure 5B, Supplemental Figure S4). After a 4 h incubation, PROP inhibited the expression of multiple genes that are known to be regulated by STAT3 in the human breast cancer cell line MDA-MB-436, further supporting the concept that PROP functions as a STAT3 inhibitor (Figure 5C).

We next sought to determine if PROP could function as a STAT3 inhibitor of peripheral blood mononuclear cells in mice with a humanized immune system. Humanized mice were treated with rescue therapy (NAC and fomepizole) alone or in combination with PROP. Thirty minutes later, the mouse was euthanized, and the blood was taken for analysis. Using ImageStream, it was shown that PROP-treated mice had fewer pSTAT3-positive CD11b^+^ cells in circulation relative to mice treated with the rescue cocktail alone (see the green boxes in Figure 5D,E; Supplemental Figure S5). These results indicate that PROP acts as a STAT3 inhibitor of peripheral blood mononuclear cells and further suggest that STAT3 phosphorylation may be a pharmacodynamic biomarker in clinical trials evaluating PROP in patients.

### 3.5. Propacetamol Has Increased Safety in Model-Dependent Fashion When Given Orally Compared to Acetaminophen

**Figure 6:**
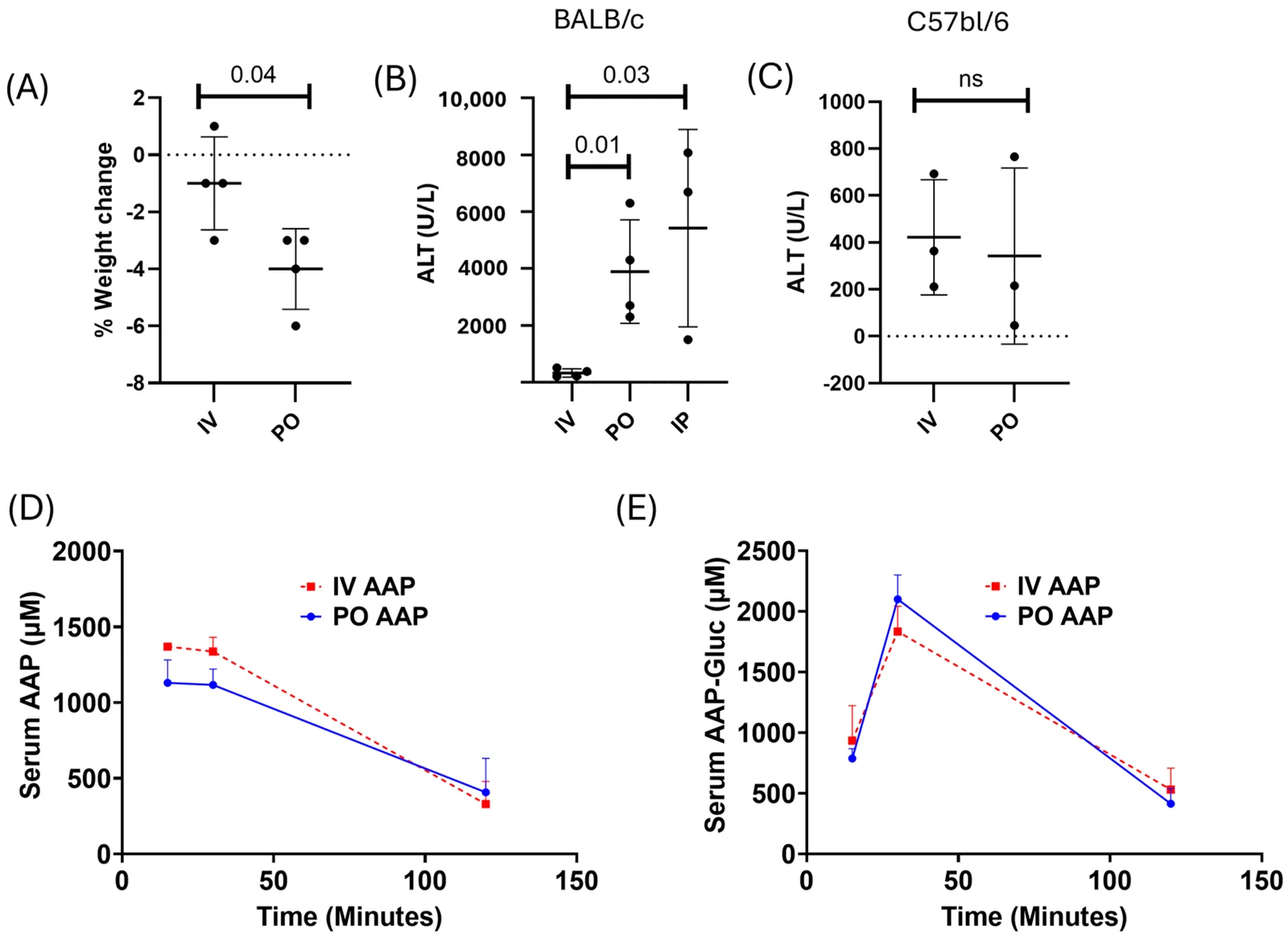
Oral acetaminophen (AAP) is more toxic than IP or IV administration in the BALB/c mouse model. (**A**) AAP was administered (250 mg/kg) via the indicated route. Body weight was measured 24 h after treatment. N = 4 mice per group. Mouse serum ALT values 1 day following administration of 250 mg/kg AAP administered orally, IV, or IP in BALBc (N = 4 mice per group for IV and PO, N = 3 mice per group for IP) (**B**) and C57bl/6 mice (N = 3 mice per group) (**C**). Results for independent mice are shown. (**D**) Mouse serum pharmacokinetics of AAP (350 mg/kg) administered orally or IV. N = 3 mice/time point/group. (**E**) Levels of the metabolite AAP-glucuronide (AAP-Gluc) were also assessed; N = 3 mice/time point/group. *p* values are shown. ns = not significant.

We next evaluated the relative safety of high-dose AAP administered orally (PO) or IV. In BALB/c mice, oral administration of AAP demonstrated greater toxicity compared to IV administration. Mice administered PO AAP exhibited significant weight loss and markedly increased ALT compared with the mice receiving IV AAP at a 250 mg/kg dose (Figure 6A,B). Interestingly, this phenomenon was only apparent in BALB/c mice but not in C57bl/6 mice; PO AAP was not more toxic in C57bl6 mice at a 250 mg/kg dose (Figure 6C). To determine if altered metabolism (such as via first-pass metabolism in the liver) may explain the difference in toxicity of PO compared to IV AAP in BALB/c mice, mass spectrometry was used to determine the kinetics of metabolite formation using established methodologies [13]. As expected, the peak serum AAP concentrations were slightly higher in the BALB/c mice receiving IV AAP compared to oral AAP. However, the concentrations of the major AAP metabolites, including AAP-Gluc, AAP-NAC and AAP-SULF, were relatively similar between the IV and PO AAP (Figure 6D,E and Supplementary Table S2). These results suggest that altered metabolic processing of AAP does not explain differences in toxicity between PO and IV administration in BALB/c mice.

**Figure 7:**
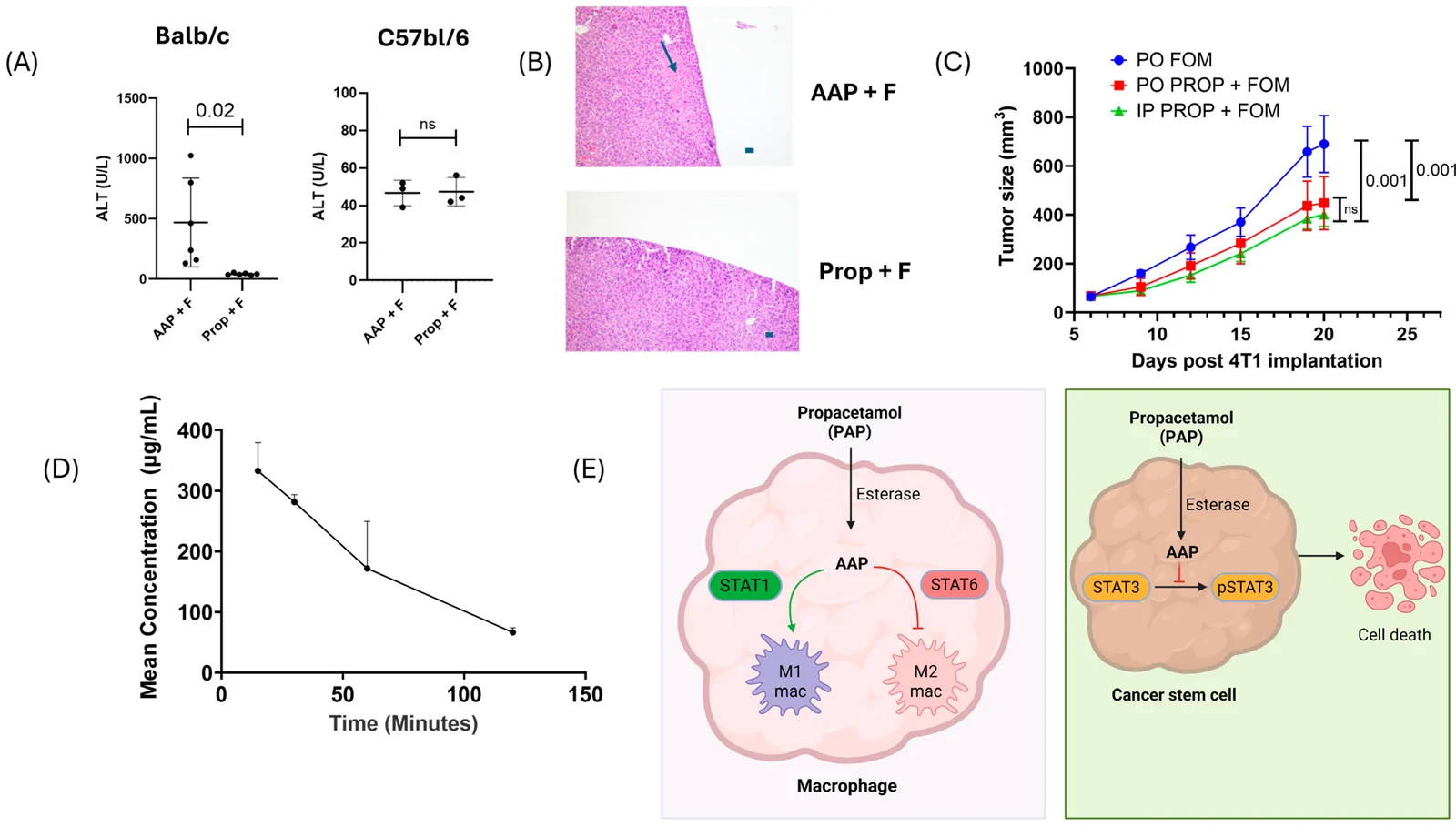
Oral propacetamol (PROP) is well tolerated and has anti-cancer activity. (**A**,**B**) Mice were treated with AAP (500 mg/kg) or PROP (1000 mg/kg) along with fomepizole (30 mg/kg), and the following day serum was analyzed (**A**) and liver histology was assessed with H&E stain with arrow pointing to necrotic area in liver of a BALB/c mouse (**B**). N = 5 mice per group in BALB/c mice and N = 3 mice per group in C57bl/6 mice. (**C**) 4T1 tumor-bearing mice were treated (average tumor size at treatment initiation: 66 mm^3^) with PROP either PO or IP (1000 mg/kg) and fomepizole (FOM, 30 mg/kg), and tumor size was monitored with digital calipers. N = 5 mice per group. (**D**) C57bl/6 mouse was treated PO with 1000 mg/kg PROP in conjunction with fomepizole, and serum was analyzed at the indicated time point for AAP concentration. N = 3 mice per time point. (**E**) Proposed mechanism of anti-cancer activity of PAP. *p* values are shown. ns = not significant.

We subsequently evaluated the safety of PO AAP in the presence of fomepizole-based rescue. Even when co-administered with fomepizole, PO AAP caused liver toxicity in BALB/c mice. However, PO PROP administered concurrently with fomepizole was well tolerated and did not cause liver toxicity (AAP given PO: ALT 470 ± 370 U/L vs. PROP given PO: 41 ± 8 U/L, *p* < 0.02, Figure 7A,B). Both high-dose PO AAP and PROP with concurrent fomepizole were well tolerated in C57bl/6 mice without liver toxicity, suggesting that the toxicity of PO AAP with fomepizole is model-dependent. While potential explanations for the differential toxicity of PO AAP in BALB/c versus C57bl6 mice are outside the scope of the present manuscript, possibilities are outlined in the Discussion below.

Due to the toxicity of PO AAP (even with fomepizole), we did not evaluate its anti-cancer activity in BALB/c models. Oral PROP, on the other hand, demonstrated antitumor efficacy that was comparable to IP administration in the 4T1 breast cancer model (final tumor size 690 ± 117 mm^3^ for vehicle, 449 ± 108 mm^3^ for PO PROP, and 401 ± 49 mm^3^ for IP PROP, Figure 7C). Oral pharmacokinetics demonstrated that oral PROP produced substantial systemic AAP exposure (measuring AAP levels because PROP levels were undetectable at all time points; Figure 7D). Key parameters for systemic exposure of AAP after PROP administration were calculated as C_max_: 332.8 µg/mL; T_max_: 0.25 h (15 min); AUC _0–2 h_: 351.4 µg·h/mL; T_1/2_ (apparent): 0.73 h (~44 min); estimated AUC_0–∞_; approximately 421.5 µg·h/mL.

A proposed mechanism of the anti-cancer activity of PAP is shown (Figure 7E). The current manuscript demonstrates enhanced intracellular delivery of AAP via PAP administration. Prior manuscripts have demonstrated effects of AAP on macrophage polarization and inhibition of cancer stem cell phenotype [5,6,14]. Direct comprehensive evaluation of PAP on macrophage phenotype and cancer stem cell proliferation (as outlined in Figure 7E) remains a future direction at this point.

## 4. Discussion

We have previously demonstrated that high doses of AAP (up to 650 mg/kg, IP) can be safely administered in conjunction with fomepizole-based rescue [4]. At these high doses, AAP exhibits anti-cancer activity against a broad range of malignancies in preclinical models [4,5,6]. However, the administration of high-dose AAP is limited by its poor water solubility, which poses challenges for formulation and delivery. PROP is an AAP pro-drug that was marketed for many years in Asia and Europe and is several-fold more water-soluble than AAP, making it possible for administration in a concentrated solution at relatively low volumes. In the present study, we evaluated the anti-cancer efficacy of water-soluble AAP pro-drugs, including PROP and an AAP phosphonate-based pro-drug. We demonstrate that PROP has greater anti-cancer activity than AAP, which appears to result from enhanced intracellular delivery of AAP in 4T1 tumor cells. While parenteral administration is the likely preferred route of administration of high-dose PROP, oral administration may provide an alternative approach. When administered orally in conjunction with fomepizole, PROP demonstrated improved safety (in a model-dependent fashion, e.g., in BALB/c mice but not C57bl/6 mice) compared to AAP and furthermore retained anti-cancer efficacy.

While not the subject of our present research, we believe that high-dose PROP administered with fomepizole-based rescue may also be a viable narcotic-sparing analgesic drug combination. The CYP2E1-mediated metabolism of AAP/PROP that is linked to toxicity and blocked by fomepizole has not been associated with AAP’s analgesic properties. While the exact mechanism underlying AAP analgesic effects remains incompletely understood, it has been postulated that fatty acid amide hydrolase (FAAH)-mediated conversion of AAP to AM404 is contributory [20]. Indeed, FAAH metabolism of AAP is not dependent on CYP2E1 and thus is unlikely to be inhibited by fomepizole. As a result, high-dose PROP with fomepizole may offer analgesic benefits; however, these studies are a future direction at this point.

The distinct toxicity profile of high-dose oral AAP in BALB/c versus C57bl/6 mice, both in the presence and absence of fomepizole rescue, is notable. We have previously demonstrated profound changes in the tumor immune microenvironment induced by high-dose AAP, thus underscoring the immune-modulating activity of the drug [5,6]. Indeed, BALB/c and C57bl/6 mice are known to have very different immune systems, with BALB/c able to mount a strong Th2-type immune response, while C57bl/6 is characterized by a strong Th1-type immune response [21]. It is reasonable to speculate that distinct immunometabolic responses can explain the distinct toxicity of oral AAP in the two mouse strains, although the details are beyond the scope of the present manuscript.

One known toxicity of PROP (distinct from AAP) is infusion-related pain that is likely related to the low pH (about 3.5) of clinically used propacetamol formulations [10]. Additional formulation work or alternative routes of administration (e.g., oral administration or IV via a central line) could help mitigate this known toxicity. Because many cancer patients have central venous access, for instance via a port, infusion-related pain may be mitigated because the low pH of PROP will presumably be better tolerated when administered centrally. Furthermore, our laboratory is actively investigating formulation strategies to improve stability of PROP at a pH closer to that of clinically used normal saline solutions (approximately pH 5.5) that are routinely administered without infusion-related pain.

PROP has several key characteristics that support its use as an AAP pro-drug, including high intracellular delivery of AAP, enhanced water solubility, stability in saline solution (albeit at relatively acidic pH), and rapid conversion to the parent AAP in vivo. Perhaps the most important characteristic is the superior antitumor efficacy PROP demonstrated relative to AAP in the 4T1 and PAN02 mouse xenograft tumor models.

The exact mechanism explaining the enhanced anti-cancer activity of PROP compared to AAP is challenging to definitively elucidate. We demonstrate increased intracellular delivery of AAP, which is a likely explanation for enhanced modulation of intracellular JAK-STAT signaling. We have previously shown that STAT3 mediates the anti-cancer stem cell activity of AAP [14]; thus, the enhanced modulation of JAK-STAT signaling likely plays a role in PROP’s increased anti-cancer activity. Furthermore, AAP inhibits STAT6-mediated M2 polarization of macrophages, which also contributes to antitumor activity; macrophage depletion reduces AAP anti-cancer activity in vivo [6]. PROP has increased inhibition of STAT6 phosphorylation compared to AAP with shorter (more physiologically relevant) incubation times. STAT3 and STAT6 promote tumor growth in PDAC and TNBC and thus are highly appealing targets in these diseases [22,23,24]. As a result, it appears reasonable to speculate that the increased intracellular delivery of AAP and resulting in more effective modulation of JAK/STAT signaling mediate the improved anti-cancer activity of PROP compared to AAP. However, we acknowledge that other possible explanations are also feasible. Experiments evaluating the efficacy of PROP in knockdown and knock-in models of STAT3 and/or STAT6 may help clarify the exact mechanisms of PROP; these studies are of ongoing interest in our laboratory. These experiments may further provide more direct evidence of potential anti-cancer stem activity of PROP; we have previously used STAT3 knockdown experiments to evaluate the role of STAT3 in mediating the anti-cancer stem cell effects of AAP [14].

Our findings exemplify the use of a pro-drug approach to enhance the physicochemical characteristics of therapeutic agents. The use of a pro-drug strategy has become increasingly common in drug development; in fact, approximately 13% of FDA-approved drugs between 2012 and 2022 were pro-drugs [25]. The present study exemplifies several of the advantages of pro-drugs that have driven widespread adoption of the approach in modern pharmacology and drug development.

## 5. Conclusions

In conclusion, our findings provide a strong preclinical rationale for advancing PROP in combination with fomepizole-based rescue into clinical evaluation for the treatment of patients with advanced malignancies.

## Data Availability

All data used in the manuscript will be made available upon request.

## Supplementary Materials

The following supporting information can be downloaded at: https://www.mdpi.com/article/10.3390/cancers18203390/s1, Figure S1: NMR reports of AAP phosphonate (A) and propacetamol (B); Figure S2: Mice with AT-3 tumors, shown in Figure 1G,H, were weighed at the conclusion of experiment; Figure S3: Phospho-AAP has decreased anti-cancer activity relative to AAP; Figure S4: 4T1, BMDM and PAN02 tumor cells were treated with IL-6 or IL-4 and indicated drugs for 30 min prior to analysis with Western blot; Figure S5: Non-tumor bearing humanized mice were treated with n-acetylcysteine (N, 100 mg/kg) and fomepizole (F, 30 mg/kg) without or with propacetamol (1000 mg/kg) or AAP (500 mg/kg); Western supplemental figures: original western blot figures; Table S1: qPCR primers; Table S2: Values of AAP metabolites at indicated timepoints after treatment as measured using mass spectrometry.
